# Keep calm and carry on: electrophysiological evaluation of emotional anticipation in the second language

**DOI:** 10.1093/scan/nsz066

**Published:** 2019-09-11

**Authors:** Rafał Jończyk, Inga Korolczuk, Evangelia Balatsou, Guillaume Thierry

**Affiliations:** 1 Faculty of English, Adam Mickiewicz University, 60-780, Poznan, Poland; 2 Department of Psychology, Pennsylvania State University, 16801, University Park, PA, USA; 3 School of Psychology, Bangor University, LL57 2AS, Bangor, UK; 4 Centre for Research on Bilingualism, Bangor University, LL57 2DG, Bangor, UK

**Keywords:** stimulus preceding negativity (SPN), anticipationserif>, emotionserif>, foreign language effectserif>, bilingualismserif>

## Abstract

Investigations of the so-called ‘foreign language effect’ have shown that emotional experience is language-dependent in bilingual individuals. Response to negative experiences, in particular, appears attenuated in the second language (L2). However, the human brain is not only reactive, but it also builds on past experiences to anticipate future events. Here, we investigated affective anticipation in immersed Polish–English bilinguals using a priming paradigm in which a verbal cue of controlled affective valence allowed making predictions about a subsequent picture target. As expected, native word cues with a negative valence increased the amplitude of the stimulus preceding negativity, an electrophysiological marker of affective anticipation, as compared with neutral ones. This effect was observed in Polish–English bilinguals and English monolinguals alike. The contrast was non-significant when Polish participants were tested in English, suggesting a possible reduction in affective sensitivity in L2. However, this reduction was not validated by a critical language × valence interaction in the bilingual group, possibly because they were highly fluent in English and because the affective stimuli used in the present study were particularly mild. These results, which are neither fully consistent nor inconsistent with the foreign language effect, provide initial insights into the electrophysiology of affective anticipation in bilingualism.

## Introduction

The human brain can be conceived as a prediction machine that builds upon prior experience to anticipate forthcoming sensory stimuli (Engel *et al.,* 2001; Bar, 2007, 2009; Van Berkum, 2010, 2013), construct emotional experiences (Barrett and Bar, 2009; Barrett and Simmons, 2015; Hoemann *et al.,* 2017) or make decisions (e.g. Summerfield and de Lange, 2014; de Lange and Fritsche, 2017). A growing body of literature suggests that the brain goes well beyond being a reactive organ merely responding to incoming stimuli, and that it constantly prepares the resources needed to increase operational efficiency (Sterling, 2012). Electrophysiological studies of anticipatory processing have investigated slow waves that build up between the presentation of a warning stimulus (S1) and a target stimulus (S2), by comparing when S1 announces S2 and when it does not (Brunia *et al.,* 2011a). In classical threat-of-shock experiments, for instance, participants expect an electrical shock (S2) on the basis of a preceding warning (S1; e.g. Böcker *et al.,* 2001). An event-related brain potential (ERP) modulation of particular interest in such S1-S2 paradigm is the contingent negative variation (CNV) (Walter *et al.,* 1964), consisting of an early phase, reflecting attention to and processing of the warning stimulus, and a late phase, reflecting S2 anticipation and motor response preparation.

When S2 is highly evocative and arousing, e.g. when it has a high affective valence, the non-motor, late phase of the CNV takes the form of a negative drift, the stimulus-preceding negativity (SPN, Kotani *et al.,* 2001; Brunia and van Boxtel, 2004; Poli *et al.,* 2007; Moser *et al.,* 2009; Brunia *et al.,* 2011b; Thiruchselvam *et al.,* 2011; Perri *et al.,* 2014; Michalowski *et al.,* 2015; Shafir *et al.,* 2015; Zheng and Liu, 2015; Pei and Meng, 2016). SPN modulations have been reported in studies manipulating the anticipation of phobia-related stimuli (e.g. Michalowski *et al.,* 2015), painful stimuli (e.g. Böcker *et al.,* 2001; Seidel *et al.,* 2015; Swannell *et al.,* 2016) or aversive noise (Kotani *et al.,* 2001).

Other studies have found an SPN amplitude increase when participants anticipate arousing, affective pictures (Takeuchi *et al.,* 2005; Poli *et al.,* 2007; Moser *et al.,* 2009; Thiruchselvam *et al.,* 2011; Perri *et al.,* 2014). For example, Poli *et al.* (2007) measured the extent to which the anticipation of negative and positive pictures would modulate SPN amplitude and heart rate. Participants were first presented with an affective (e.g. ‘blood’) or neutral (e.g. ‘people’) verbal cue preparing them for upcoming pictures of varying valence (positive, negative and neutral) and arousal (high arousal and low arousal). Irrespective of affective valence, SPN amplitudes increased and heart rate slowed down in the anticipation of highly arousing pictures (e.g. erotic content or injuries) relative to low arousing pictures, even though Poli *et al.* (2007) used only six words as S1 cues. Swannell *et al.* (2016) also found that words associated with the concept of pain modulated participants’ anticipation of a heat stimulation delivered through a laser, when S1 presentation was subliminal, i.e. unconscious.

Here, we set out to investigate whether affective anticipation can be modulated by the language in which S1 is presented, within the same participants, and when S1 valence is matched between languages, in order to test the hypothesis of dampened emotional response when imbalanced bilinguals operate in their second language (Wu and Thierry, 2012; Jończyk, 2016a; Jończyk *et al.,* 2016; Sheikh and Titone, 2016; Baumeister *et al.,* 2017; Iacozza *et al.,* 2017; Jankowiak and Korpal, 2017).

For instance, in a recent eye-tracking study, Iacozza *et al.* (2017) reported increased pupil dilation, indexing physiological arousal, in 27 Spanish–English bilinguals reading sentences with a negative connotation in Spanish, their native language (L1), as compared with English, their second language (L2). In the same vein, Jankowiak and Korpal (2017) asked 27 Polish–English bilinguals to listen to or read fragments that were negative (e.g. narrating someone’s death) or neutral (e.g. describing a city) in Polish or in English. When comparing English to Polish contexts, the authors reported decreased electrodermal activity in participants reading negative descriptions, but this was only found for reading as opposed to listening to the descriptions. These findings corroborate earlier electrophysiological evidence for reduced amplitudes of the N400, an electrophysiological index of semantic processing, selectively when negative information was presented in L2, whether in word pairs (Wu and Thierry, 2012) or naturalistic sentences (Jończyk *et al.,* 2016). Thus, operating in the non-native language appears to provide late bilinguals with some kind of affective protection vis-à-vis negative emotional content. These findings provide neurophysiological support for bilinguals’ subjective reports about their emotional experiences in L1 and L2 in which the bilinguals’ L2 is often portrayed as being emotionally more distant, disembodied, or detached from early memories compared with their L1 (for reviews, see Pavlenko, 2012, Caldwell-Harris, 2015 and Jończyk, 2016a).

Other studies have reported effects consistent with the above reviewed results but in terms of a relatively greater sensitivity to positive information in L1, which can be considered the mirror image of reduced sensitivity to negativity in L2. For example, Hsu *et al.* (2015) observed increased hemodynamic response to positive extracts from Harry Potter when these were presented to 24 German–English bilinguals in their L1 as compared with their L2. Gao *et al.* (2015) further showed that positive feedback delivered in Chinese to 16 Chinese–English bilinguals incited them to take 10% more risk in the following even-probability gambling trial, irrespective of amounts to be won or lost, whereas no difference was observed between positive and negative conditions when feedback was provided in English. Overall, despite the initial null results reported in electrophysiological studies of emotion-language interaction in bilinguals (Conrad *et al.,* 2011; Opitz and Degner, 2012), the evidence accumulated so far sways in favor of a moderating effect of the second language on emotional experience in late bilinguals.

However, previous studies showing dampened affective sensitivity in L2 stand in contrast to results from cognitive studies using behavioral paradigms such as the emotional Stroop task (Sutton *et al.,* 2007; Eilola *et al.,* 2007; Grabovac and Pléh, 2014; but see Winskel, 2013), the affective priming task (Degner *et al.,* 2012) or the emotional word recall and recognition task (Ferré *et al.,* 2010; Ferré *et al.,* 2013; but see Baumeister *et al.,* 2017). Indeed, the latter studies have reported little or no difference in the automaticity of emotional word processing across L1 and L2 (for most recent reviews, see Caldwell-Harris, 2015; Jończyk, 2016a).

In this study, we examined, for the first time, the neurophysiological effects of language of operation on anticipatory affective processing, that is how the bilingual brain prepares for an upcoming emotional event. We employed the S1–S2 paradigm commonly implemented in studies of anticipation in combination with ERPs to investigate the relative amplitude of anticipatory potential variations elicited by stimulus cues (see Wu and Thierry, 2017, for a similar approach in the context of preparation for speaking). To our knowledge, only one study has previously investigated a possible interaction between affective anticipation and language of operation using physiological measures (García-Palacios *et al.,* 2018). However, that study focused on language use (counting task), not semantic processing, and targeted physiological measures that are a proxy for stress levels in a fear conditioning context (pupil dilation and electrodermal activity).

In the present experiment, participants were presented with a negative, positive or neutral verbal cue (S1), either in their L1 (Polish) or in their L2 (English). The cue predicted the valence and meaning of the target picture (S2) in 50% of trials. Following picture presentation, participants were asked to determine whether the target picture and verbal cue were related in meaning or not. Consistent with previous SPN studies (van Boxtel and Böcker, 2004; Moser *et al.,* 2009; Ohgami *et al.,* 2014; Shafir *et al.,* 2015), we focused on an early and a late time window of analysis to obtain a comprehensive picture of the anticipatory period preceding S2 presentation. We focused predictively on the relative impact of negative compared with neutral cues when late Polish–English bilinguals anticipated the display of emotional pictures, since positive stimuli usually afford less sensitivity.

Consistent with a hypothetical mechanism of repression (Wu and Thierry, 2012), we predicted more negative SPN amplitudes in both the early and late time windows of the SPN following a negative *vs* a neutral cue in L1 Polish but no such modulation for L2 English cues. We also recruited a group of monolingual English controls to ensure that the English stimuli would successfully elicit affective anticipation effects in native speakers of the language and thus expected to find SPN modulations in both the early and the late time windows in the group of native English speakers tested in English.

## Methods

### Participants

Twenty-one Polish–English bilinguals and twenty-one English monolingual speakers from the area of North Wales gave informed consent to take part in the study that was approved by the ethics committee of Bangor University, Wales, UK. Data from four bilingual and three monolingual participants were discarded due to insufficient number of clean segments of electrophysiological (EEG) data per condition or excessive alpha contamination. This resulted in a final participant sample of 17 bilinguals (*M*_age_ = 23.4, standard deviation [SD] = 3.9; 6 males, 11 females) and 18 monolinguals (*M*_age_ = 24.4, SD = 2.7; 12 males, 6 females). Except one ambidextrous monolingual participant, participants from both groups were right-handed. Also, all participants were residing in North Wales at the time of the experiment and reported having (corrected-to-) normal vision.

The bilingual participant group consisted of Polish native speakers who acquired English after puberty. All participants were immersed in the British culture at the time of testing, but immersion time varied widely, from 3 months to 16 years (*M*_immersion_ = 6.6 years, SD = 5.8). Constraining participants selection on the basis of cultural exposure was not possible given the relatively small size of the Polish community in North Wales. The participants reported using both Polish and English on everyday basis, in both formal and informal contexts. Their English proficiency in reading, writing, speaking and listening was self-reported using an adapted version of the Language History Questionnaire 2.0 (Li *et al.,* 2014; Table 1). The control monolingual participant group consisted of native English speakers who acquired English after birth and had limited exposure to other languages. Participants were compensated for their time with £12.

**Table 1:** Bilingual participants’ self-rated proficiency in English

Measure	Score
Overall	7.75 [1.2]
Reading	7.83 [1.9]
Writing	7.52 [1.7]
Speaking	7.52 [1.5]
Listening	7.82 [1.1]

Proficiency was measured with a 10-point Likert scale where 1 = very poor, 10 = native-like. Square brackets represent s.d.m.

### Stimuli

The experiment conducted with bilinguals comprised of 62 positive, 62 negative and 124 neutral English words selected from an affective word database (Warriner *et al.,* 2013), paired with 62 positive and 62 negative pictures taken from Google image online databases. The word-picture pairs were related or unrelated in meaning based on a pre-experimental norming study. Each word served as a cue to elicit anticipation of a subsequently presented picture in four affective cue-target combinations: negative word-negative picture, positive word-positive picture, neutral word-negative picture and neutral word-positive picture. In each case, 62 combinations were related and 62 were unrelated. Unrelated pairs were created by repairing word cues and pictures within the positive and negative conditions and making sure through pre-experimental norming that no spurious relatedness randomly arose. Polish word cues were translations of the English word cues implemented by a highly proficient Polish–English bilingual with expertise in bi-directional Polish–English translation. To validate stimulus selection, all items were back-translated from Polish to English by six proficient Polish–English bilinguals who did not take part in the study: 85% of translations overlapped three or more times out of six. The remaining 15% yielded fewer stable translations, but most were closely related or synonymous (e.g. ‘rich’ for ‘wealthy’, ‘tumor’ for ‘cancer’, ‘baby’ for ‘new-born’, etc.). Inter-rater agreement between translators was high (Intraclass Correlation Coefficient, ICC = 0.84).

Zipf values for word lexical frequencies were collected from SUBTLEX-UK (van Heuven *et al.,* 2014) and SUBTLEX-PL (Mandera *et al.,* 2015) databases. In English, no significant differences were observed in lexical frequencies between positive (*M* = 4.38; 95% confidence interval [CI] [4.21, 4.53]), negative (*M* = 4.17; 95% CI [4.0, 4.32]) and neutral (*M* = 4.32; 95% CI [4.20, 4.43]) words, *F*_(2,245)_ = 2.05, *P* = 0.13, η^2^ = 0.01. Similarly, positive (*M* = 3.9; 95% CI [3.74, 4.06]), negative (*M* = 3.68; 95% CI [3.65, 3.84]) and neutral (*M* = 3.77; 95% CI [3.65, 3.88]) words in Polish did not significantly differ in lexical frequency, *F*_(2,245)_ = 1.78, *P* = 0.17, η^2^ = 0.01; however, Polish words (*M* = 3.78; 95% CI [3.7, 3.87]) were overall slightly less frequent than English words (*M* = 4.29; 95% CI [4.20, 4.37]), *F*_(1,494)_ = 78.1, *P* < 0.001, η^2^ = 0.13.

**Fig. 1:**
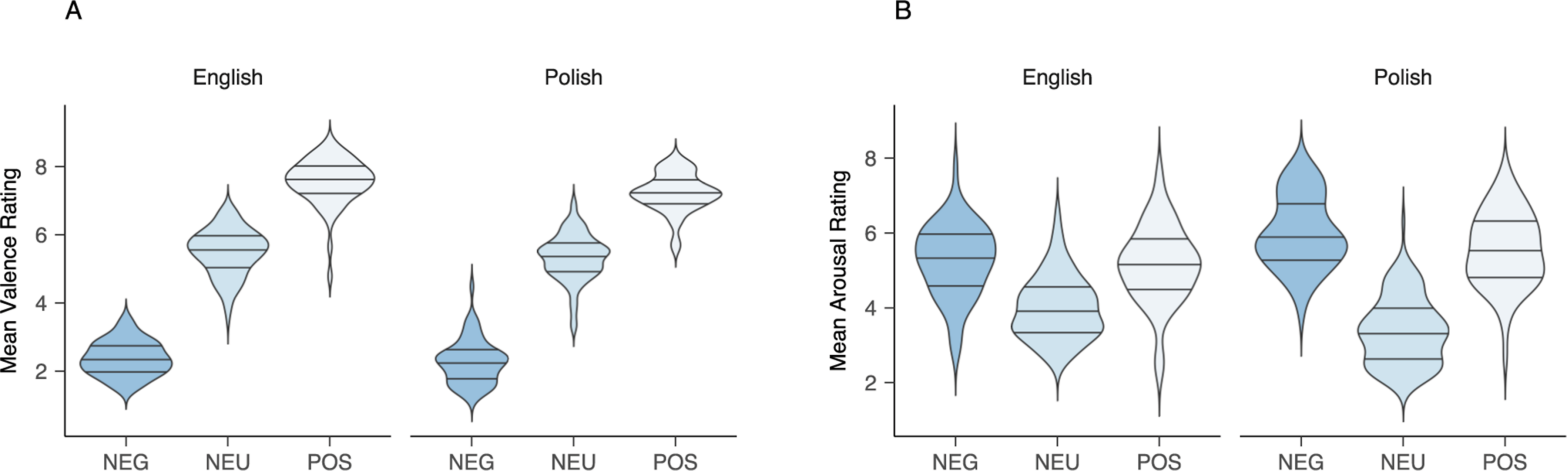
Violin plots of valence (A) and arousal (B) ratings for English and Polish word cues. Shapes represent density estimates and horizontal lines represent inter-quartile range (25, 50 and 75%). NEG, words with negative valence words, NEU, neutral words, and POS, words with positive valence.

Ratings of valence and arousal for English words were obtained from Warriner *et al.* (2013). Valence differed significantly between positive (*M* = 7.56; 95% CI [7.4, 7.72]), negative (*M* = 2.37; 95% CI [2.21, 2.53]) and neutral (*M* = 5.48; 95% CI [5.37, 5.60]) words, *F*_(2,245)_ = 1048, *P*_s_ < 0.001, η^2^ = 0.89. Arousal differed only between positive (*M* = 5.15; 95% CI [5.02, 5.49]) and neutral (*M* = 4.0; 95% CI [3.83, 4.16]; *P*_bonf_ < 0.001) as well as negative (*M* = 5.26; 95% CI [5.02, 5.49]) and neutral words, *P*_bonf_ < 0.001), with no difference between positive and negative words (*P*_bonf_ = 0.1). Based on post-experimental valence norming reported in a previous experiment with the same population (Jończyk, 2016b; Jończyk *et al.,* 2016) and the fact that Polish words were direct translations of the English words, we assumed rough comparability in valence and arousal between languages. Nevertheless, we tested this assumption post-experimentally with a group of Polish speakers who did not take part in the experiment. The 248 Polish words used in the experiment were rated on valence and arousal on a scale from 1 to 9 (1 = highly negative/low arousing; 9 = highly positive/highly arousing) by 29 and 26 individuals, respectively. The scale and rating procedures followed Warriner *et al.* (2013), to ensure comparability between scales and instructions for further analysis. The scores obtained from this study and the database by Warriner *et al.* (2013) were subjected to two 3 (valence: positive; negative; neutral) × 2 (language: Polish; English) by-item ANOVAs. The emotionality analysis revealed a main effect of valence, *F*_(2,490)_ = 1982, *P* < 0.001, η^2^ = 0.89, with significant differences between negative (*M* = 2.32; 95% CI [2.20, 2.43]), positive (*M* = 7.39; 95% CI [7.28, 7.50]) and neutral (*M* = 5.4; 95% CI [5.32, 5.48]) words, *P*_s_ < 0.001. The valence × language interaction was non-significant, *F*_(2,490)_ = 1.15, *P* = 0.30, η^2^ = 0.01. The arousal analysis revealed a main effect of valence, *F*_(2,490)_ = 236.24, *P* < 0.001, η^2^ = 0.49, with higher arousal ratings for negative (*M* = 5.63; 95% CI [5.47, 5.79]) and positive (*M* = 5.35; 95% CI [5.19, 5.52]) words relative to neutral words (*M* = 3.6; 95% CI [5.19, 5.52]; *P*_s_ < 0.001). Furthermore, negative words were rated as marginally more arousing than positive words, at *P* = 0.06. Finally, the arousal analysis revealed a significant valence × language interaction, *F*_(2,490)_ = 26.95, *P* < 0.001, η^2^ = 0.09, with higher arousal ratings for negative and positive words in Polish (*M*_negative_ = 6.01, 95% CI [5.77, 6.24]; *M*_positive_ = 5.56, 95% CI [5.32, 5.79]) relative to English (*M*_negative_ = 5.26, 95% CI [5.02, 5.49]; *M*_positive_ = 5.15, 95% CI [4.92, 5.38]), at *P*_s_ < 0.001. This effect was reversed for neutral words, with higher arousal ratings for neutral words in English (*M* = 4.0, 95% CI [3.83, 4.16]) relative to neutral words in Polish (*M* = 3.37, 95% CI [3.21, 3.54]), at *P* = 0.02. Note, however, that ratings for Polish stimuli were obtained from a group of Polish native speakers, while ratings for English stimuli were obtained from the database by Warriner *et al.* (2013); hence, small differences across ratings were expected. For a graphical representation of valence and arousal ratings, see Figure 1.

Prior to the experiment, 174 participants rated 496 word-picture pairs for semantic relatedness on a scale from 1 (completely unrelated) to 7 (highly related). Unrelated pairs consisted of word-picture pairings in which the meaning of a word was not reflected in a picture; related pairs consisted of word-picture pairings in which the meaning of a word was reflected in a picture. To avoid word repetition, the pairs were counterbalanced across participants so that each verbal cue appeared only once in each version of the norming study. This resulted in four different versions of the norming study, each containing 124 word-picture pairs. A 3 (cue: positive, neutral, negative) × 2 (relatedness: related, unrelated) by-item ANOVA was run, with valence as a between-item factor, to assess potential differences in verbal cue-picture relatedness between pairs of varying affective valence. A main effect of relatedness was found, whereby related word-picture pairs were rated as more related (*M* = 6.03, 95% CI [5.94, 6.12]) than unrelated (*M* = 2.01, 95% CI [1.92, 2.10]) word-picture pairs (*F*_(1,490)_ = 3648.8, *P* < 0.001, η^2^ = 0.87). The interaction between word cue valence and relatedness did not reach significance, *F*_(2,490)_ = 0.65, *P* = 0.52, η^2^ = 0.0, indicating that positive, negative and neutral words did not significantly differ with regard to relatedness to target pictures.

Finally, 38 participants rated 124 pictures on two affective qualities: valence and arousal. Valence was rated on a scale from −3 (very negative) to +3 (very positive) and arousal on a scale from 1 (neutral) to 5 (very arousing). A one-way by-item ANOVA demonstrated a significant main effect of valence whereby negative pictures were rated as significantly more negative (*M* = −1.65, 95% CI [−1.78, −1.50]) than positive pictures (*M* = 1.69, 95% CI [1.55, 1.80]; *F*_(1,122)_ = 1134, *P* < 0.001, η^2^ = 0.90). A second one-way by-item ANOVA found a significant main effect of arousal, with negative pictures rated as more arousing (*M* = 3.17, 95% CI [3.05, 3.29]) than positive ones (*M* = 2.88, 95% CI [2.75, 2.99]; *F*_(1,122)_ = 11.1, *P* = 0.001, η^2^ = 0.08). Overall, however, both positive and negative pictures were found to be moderate in terms of arousal. Finally, for the purposes of the experiment, the selected pictures were re-shaped in Adobe Photoshop CS6 to be 377 × 377 pixels, corresponding to a viewing angle of 5°.

### Procedure

Participants were seated 100 cm away from a cathode-ray tube (CRT) monitor in a dimly lit and quiet room. Following EEG cap preparation, during which they completed a short questionnaire, participants were familiarized with the task. They were told that in each trial they would see a verbal cue announcing a picture target. In half of the trials (124 per language), the verbal cue was affectively neutral and was followed by either a positive or a negative picture target. Thus, in this condition, the cue did not allow strong anticipatory effects to take place. In the other half of the trials, the verbal cue was positive (62 per language) or negative (62 per language), and it was followed by a picture target of the matching affective valence. Thus, when the cue was affectively arousing, it enabled affective anticipation to take place. Following a word cue, target pictures were either related (50%) or unrelated (50%). For instance, the word cue ‘accident’ could be followed either by a picture depicting a motorbike accident or by the picture of a slave. Participants were asked to indicate whether the picture was related to the preceding verbal cue or not by pressing one of two designated buttons. Participants were not explicitly informed about the emotional relationship between cue words and pictures.

The structure of a trial was as follows: the word cue announcing a target was presented for a random duration between 300 and 400 ms (in steps of 10 ms), followed by a fixation cross that remained on the screen for 3800 ms—the anticipation window. Subsequently, a picture was flashed for 200 ms and participants had to respond within 2300 ms. After every 10 trials, a pink fixation cross was displayed for 5000 ms during which time participants could rest their eyes and blink as needed. Across the experimental session, each picture was presented twice to English native speakers and four times to Polish–English bilinguals. As for word cues, they were never repeated within language in English native speakers or across languages in Polish–English bilinguals.

Bilingual participants completed two blocks in English and two blocks in Polish. Languages were alternated between blocks; block order and response sides were counterbalanced between participants. Monolingual participants completed only two blocks of trials in English. Two researchers, one native speaker of Polish and one native speaker of English were present at all times, enabling a short exchange in the language of the forthcoming block after each pause.

### EEG recording and analyses

Electrophysiological data were continuously recorded in reference to electrode Cz at a rate of 1000 Hz from 64 Ag/AgCl electrodes placed according to the extended 10–20 convention. The vertical and horizontal electrooculograms (EOGs) were recorded from electrodes located above and below the right eye and at the outer canthus of each eye. Impedances were kept below 5 kΩ. EEG signals were amplified with Neuroscan SynAmps2 amplifier unit (El Paso, TX) and filtered online with a band pass filter between 0.05 and 200 Hz.

All pre-processing steps and analyses were performed using EEGLAB Toolbox (version 14.1.1; Delorme and Makeig, 2004) and ERPLAB Toolbox (version 6.1.4; Lopez-Calderon and Luck, 2014) in MATLAB (version R2017a, The Mathworks, Inc.). The signals were down-sampled offline to 250 Hz and all data were visually inspected for abnormalities. Sections of continuous data containing gross muscle artefacts were rejected. Abnormal channel activity was identified using the trimOutlier plugin (Lee and Miyakoshi SCCN, INC, UCSD) as well as by plotting channel spectra and maps in EEGLAB. No more than four channels were rejected per dataset in both the bilingual (*M* = 1.32; min = 0, max = 4) and monolingual (*M* = 0.35; min = 0, max = 2) participant groups. The rejected channels were interpolated using the spherical spline interpolation method. Next, continuous (non-segmented) data were digitally re-referenced to the average of all scalp electrodes (global average reference), excluding EOGs, and band-pass filtered between 0.1 and 30 Hz using a second-order infinite impulse response Butterworth digital filter (slope: 12 dB/oct). Subsequently, the extended infomax independent component analysis (ICA; Lee *et al.,* 1999) was run to correct for vertical and horizontal EOG artefacts. The independent components (ICs) were inspected using a semi-automated procedure. First, the IClabel plugin (Pion-Tonachini *et al.*, 2019) was run to automatically classify ICs into broad source categories. Subsequently, selected ICs corresponding to artefactual activity originating in the eyes were visually inspected by plotting component activations. The mean number of rejected ICs per participant amounted to 1.47 (SD = 0.71; min = 1, max = 3) in the bilingual and 1.66 (SD = 0.59; min = 1, max = 3) in the monolingual group. Prior to accepting ICA correction, we plotted the EEG data before and after ICA correction to make sure that rejecting ICs did not impact the data in an adverse way.

Following ICA correction, epochs were extracted from the continuous EEG. For SPN analysis, 4000 ms epochs were extracted, starting 200 ms before anticipation cue onset. For the N400 analysis, 1000 ms epochs were extracted, starting 200 ms before target picture onset. Baseline correction was applied relative to pre-stimulus activity. Epoch rejection was based on the result of a peak-to-peak moving window in ERPLAB (threshold: ±80 μV; window size: 200 ms; window step: 100 ms) and subsequent visual inspection. Tables 2 and 3 present the mean number of accepted epochs per condition for the SPN (max = 62) and N400 (max = 124) analyses, respectively, in each participant group.

**Table 2:** The mean number of accepted epochs per condition for the SPN analysis in each group

Condition	Bilingual	Monolingual
Negative English	58.8 [57.6, 60.1]	58.8 [56.6, 60.9]
Positive English	59.0 [57.6, 60.4]	57.8 [55.3, 60.3]
Neutral English	58.8 [57.6, 60.0]	56.8 [54.0, 59.4]
Negative Polish	58.9 [57.7, 60.1]	N/A
Positive Polish	59.3 [58.3, 60.2]	N/A
Neutral Polish	59.1 [57.9, 60.3]	N/A

Square brackets represent 95% CI values.

**Table 3:** The mean number of accepted epochs per condition for the N400 analysis in each group

Condition	Bilingual	Monolingual
Related English	96.1 [90.5, 101.7]	91.9 [86.5, 97.3]
Unrelated English	103.1 [97.6, 108.6]	95.5 [88.1, 103]
Related Polish	96.9 [92.1, 101.8]	N/A
Unrelated Polish	104.5 [100.2, 108.8]	N/A

Square brackets represent 95% CI values.

We focused on two ERP components: SPN, indexing anticipatory processes, and N400, indexing cue-picture integration; the ERP components were analyzed predictively based on prior findings. SPN was analyzed over three centrofrontal midline electrodes (AFZ, FCz and Fz) in two temporal windows, 800–1200 ms post-cue (early SPN) and 300 ms prior to picture presentation (late SPN; e.g. Moser *et al.,* 2009); N400 was analyzed over nine electrodes (FC1, FCz, FC2, C1, Cz, C2, CP1, CPz and CP2) in the 350–500 ms time window (see Kutas and Federmeier, 2011).

Statistical analyses were conducted on mean SPN amplitudes in early (800–1200 ms) and late (3500–3800 ms) time window of predicted maximal sensitivity for early and late SPN, respectively. We first conducted repeated measures (RM) ANOVA on the SPN mean amplitudes in the bilingual group, with language (Polish, English) and valence (negative, neutral) as within-subject independent variables (we left the positive valence condition aside, given its commonly acknowledged reduced ability to elicit reliable SPN modulations). We then used planned comparisons by means of two-tailed paired *t*-tests to test the following predictions. (i) SPN amplitude should differ between negative and neutral anticipation conditions in Polish participants processing words in Polish. (ii) The same contrast should be reduced in amplitude or canceled when Polish participants are tested with English word cues. (iii) SPN amplitude should differ between negative and neutral anticipation conditions in English participants processing words in English. Furthermore, (iv) there should be an overall difference in SPN amplitude between English and Polish version of the experiment in Polish–English bilinguals, with more negative amplitude when testing was conducted in Polish than in English (Wu and Thierry, 2017). Note that we did not conduct a between-group comparison in this study because the conditions under which the two groups were tested varied in several ways (bilinguals knew two languages as opposed to only one, they underwent a double session comprising four blocks rather than two, they saw four instances of each picture rather than two and they had to switch between languages across experimental blocks) making such a comparison invalid. Thus, English native speakers in this study were considered a control group to independently validate the English stimuli. Finally, (v) we predicted a significant modulation of N400 amplitude elicited by picture targets when comparing unrelated *vs* related cue-picture pairs, irrespective of group or language, but we had no prediction as regards the direct comparison of Polish and English cue-picture pairs in Polish–English bilinguals. N400 analyses were performed on correct trials only by means of RM ANOVA with relatedness (related, unrelated), valence (negative, neutral) and language (Polish, English) as within-subject independent variables.

## Results

### Polish–English bilinguals

***SPN***. In the early SPN time window, the RM ANOVA revealed a main effect of valence, *F*(1,16) = 7.6, *P* = 0.01, ${\eta}_{\mathrm{p}}^2$ = 0.32, with more pronounced SPN amplitudes following negative (*M* = −1.0 μV, 95% CI [−1.7, −0.3]) rather than neutral (*M* = −0.6 μV, 95% CI [−1.4, 0.2]) word cues. Also, there was a main effect of language, *F*(1,16) = 5.2, *P* = 0.04, ${\eta}_{\mathrm{p}}^2$ = 0.24, with more pronounced SPN amplitudes following word cues in English (*M* = −1.1 μV, 95% CI [−1.9, −0.3]) as compared with Polish (*M* = −0.5 μV, 95% CI [−1.4, 0.3]). The language × valence interaction did not reach the significance threshold, *F*(1,16) = 1.8, *P* = 0.2, ${\eta}_{\mathrm{p}}^2$ = 0.10.

**Fig. 2:**
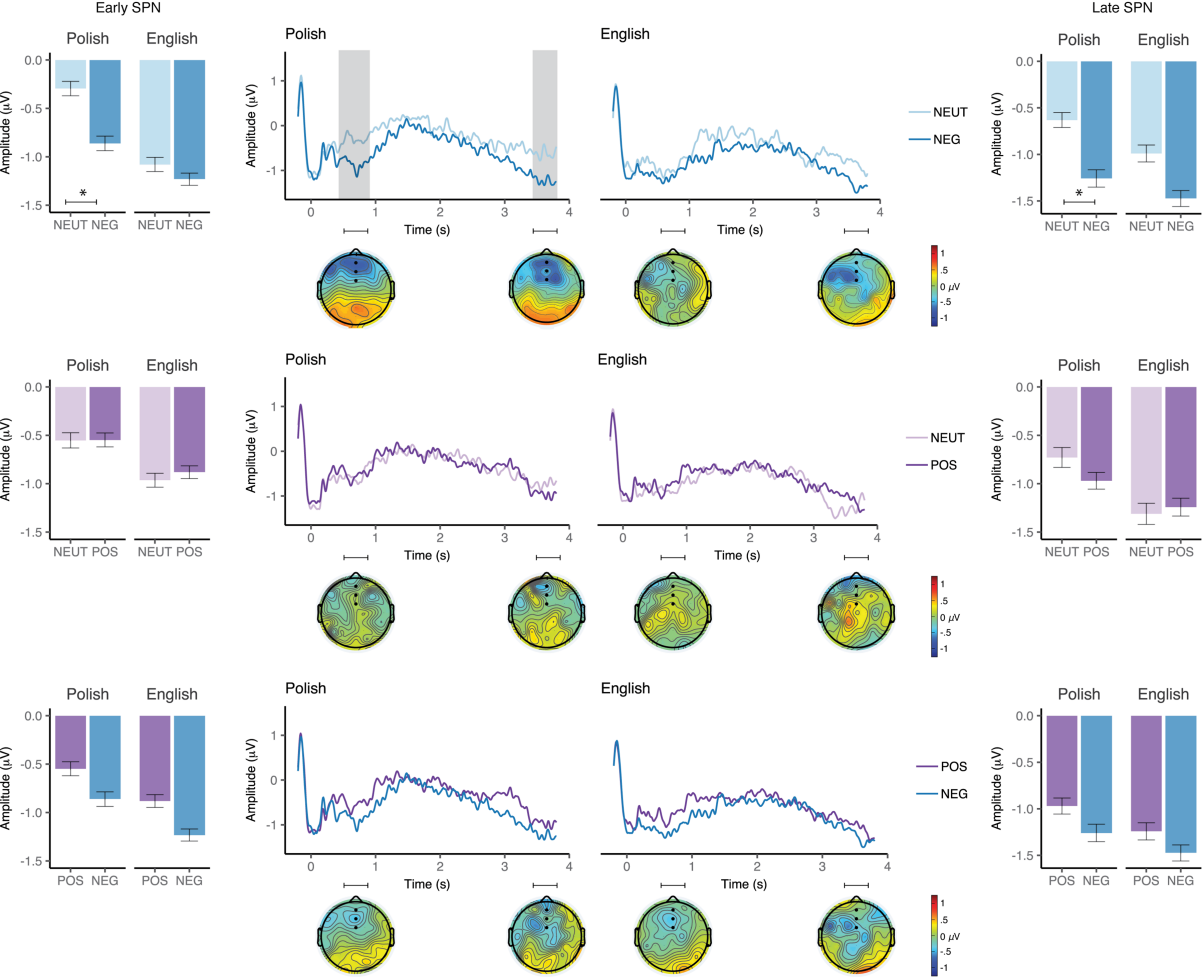
Event-related potentials elicited by neutral and negative (top), neutral and positive (middle) and positive and negative (bottom) L1 Polish (left) and L2 English (right) cues in the bilingual group. Waveforms illustrate brain potential variations computed via linear derivation from three midline frontal electrodes (AFZ, FZ and FCZ). The window of analysis started just after verbal cue presentation, coinciding with the display of a fixation cross. The shaded areas highlight the window (s) of significant differences. Black dots on the topographical maps depict the electrode sites of interest. Bar graphs represent mean SPN amplitude averaged over the electrodes of interest in each condition in the early (left panel) and late (right panel) SPN window. Error bars depict 95% confidence interval.

In the late SPN time window, the RM ANOVA revealed a main effect of valence, *F*(1,16) = 7.3, *P* = 0.02, ${\eta}_{\mathrm{p}}^2$ = 0.31, with more increased SPN amplitudes following negative (*M* = −1.3 μV, 95% CI [−2.2, –0.4]) rather than neutral (*M* = −0.77 μV, 95% CI [−1.5, 0.0]) word cues. Neither the main effect of language, *F*(1,16) = 1.1, *P* = 0.3, ${\eta}_{\mathrm{p}}^2$ = 0.1, nor the language × valence interaction, *F*(1,16) = 0.3, *P* = 0.5, ${\eta}_{\mathrm{p}}^2$ = 0.02, turned out to be significant.

**Fig. 3:**
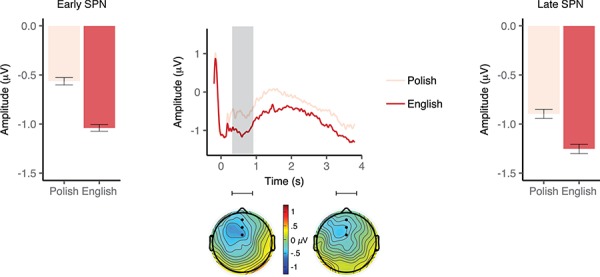
Event-related potentials elicited by cue language in the bilingual group. Waveforms illustrate brain potential variations computed via linear derivation from three midline frontal electrodes (AFZ, FZ and FCZ). The window of analysis starts just after verbal cue presentation, coinciding with the display of a fixation cross. The shaded area highlights the window of significant differences. Black dots on the topographical maps depict the electrode sites of interest. Bar graphs represent mean amplitude averaged over the electrodes of interest in the early (left panel) and late (right panel) SPN window. Error bars depict 95% confidence interval.

In the planned comparisons analyses, we found a significant modulation of the SPN amplitude in the early SPN window, with more pronounced SPN amplitudes in the anticipation of a picture cued by a negative (*M* = −0.82 μV, 95% CI [−1.7, 0.07]) as compared with a neutral (*M* = −0.26 μV, 95% CI [−1.14, 0.63]) word in Polish, *t*(16) = −2.4, *P* = 0.03, Cohen’s *d* = 0.58, but not in English, *t*(16) = −0.86, *P* = 0.40, Cohen’s *d* = 0.20. A similar result was found in the late SPN window, with more pronounced SPN amplitudes in the anticipation of a picture cued by a negative (*M* = −1.2 μV, 95% CI [−2.2, −0.26]) as compared with a neutral (*M* = −0.59 μV, 95% CI [−1.38, 0.20]) word in Polish *t*(16) = −2.5, *P* = 0.02, Cohen’s *d* = 0.61, but not in English, *t*(16) = −1.9, *P* = 0.08, Cohen’s *d* = 0.46 (see, Figures 2 and 3).

**Fig. 4:**
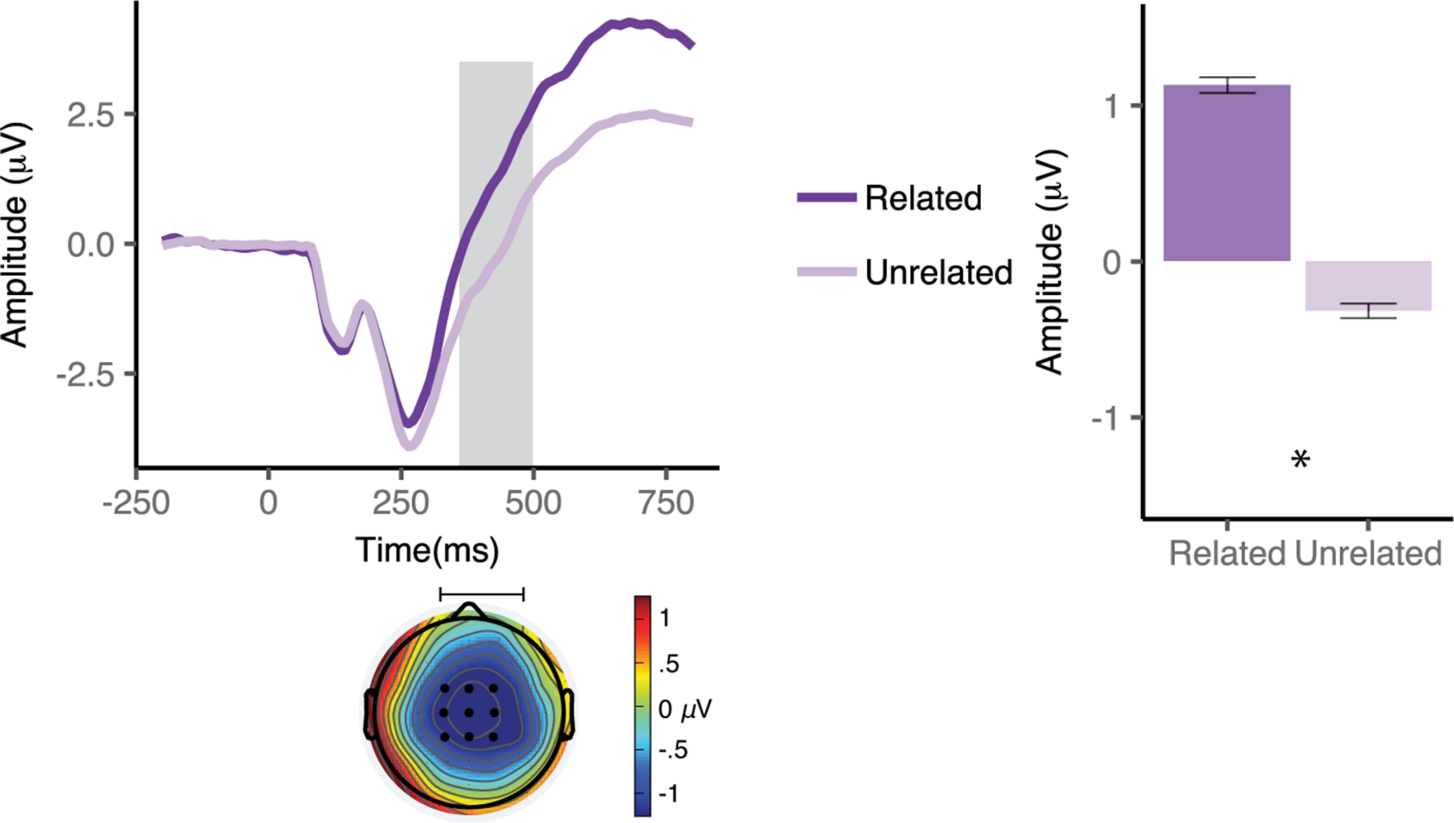
ERPs elicited by picture targets (S2) as a function of relatedness with the preceding verbal cue (S1) in the bilingual group. Waveforms illustrate brain potential variations computed via linear derivation from nine centro–parietal electrodes (FC1, FCz, FC2, C1, Cz, C2, CP1, CPZ and CP2). Time 0 coincides with picture presentation onset. The shaded area highlights the window of significant differences. Black dots on the topographical maps depict the electrode sites of interest. The bar graph represents mean N400 amplitude averaged over the electrodes of interest in the window of analysis. Error bars depict 95% confidence interval.

**Fig. 5:**
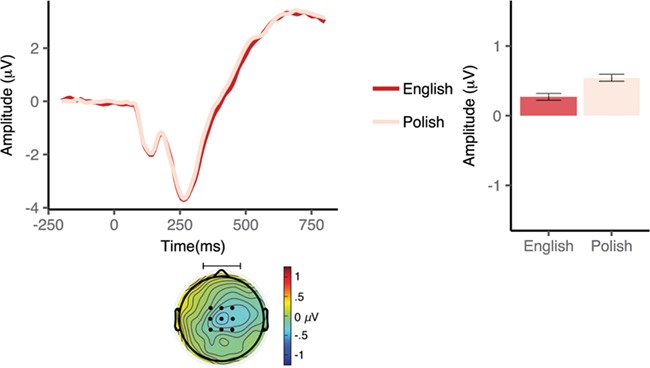
ERPs elicited by picture targets (S2) as a function of the language (L1 Polish or L2 English) of the verbal cue (S1) in the bilingual group. Waveforms illustrate brain potential variations computed via linear derivation from nine centro–parietal electrodes (FC1, FCz, FC2, C1, Cz, C2, CP1, CPZ and CP2). Time 0 coincides with picture presentation onset. Black dots on the topographical maps depict the electrode sites of interest. The bar graph represents mean N400 amplitude averaged over the electrodes of interest in the window of analysis. Error bars depict 95% confidence interval.

***N400***. The RM ANOVA revealed a significant main effect of relatedness, *F*(1,16) = 46.5, *P* < 0.001, ${\eta}_{\mathrm{p}}^2$ = 0.7, the N400 being more negative in response to pictures unrelated to the cue, *M* = −0.3 μV, 95% CI [−1.4, 0.8], than those related to the cue, *M* = 1.1 μV, 95% CI [−0.2, 2.3] (Figure 4). N400 topography was asymmetric, with slightly greater amplitudes over the right hemisphere. Furthermore, neither the main effect of language, *F*(1,16) = 0.9, *P* = 0.35, ${\eta}_{\mathrm{p}}^2$ = 0.06 (see, Figure 5), nor the main effect of valence, *F*(1,16) = 1.5, *P* = 0.26, ${\eta}_{\mathrm{p}}^2$ = 0.08, nor any of the interactions turned out to be statistically significant (*P* > 0.05).

### English monolinguals

**Fig. 6:**
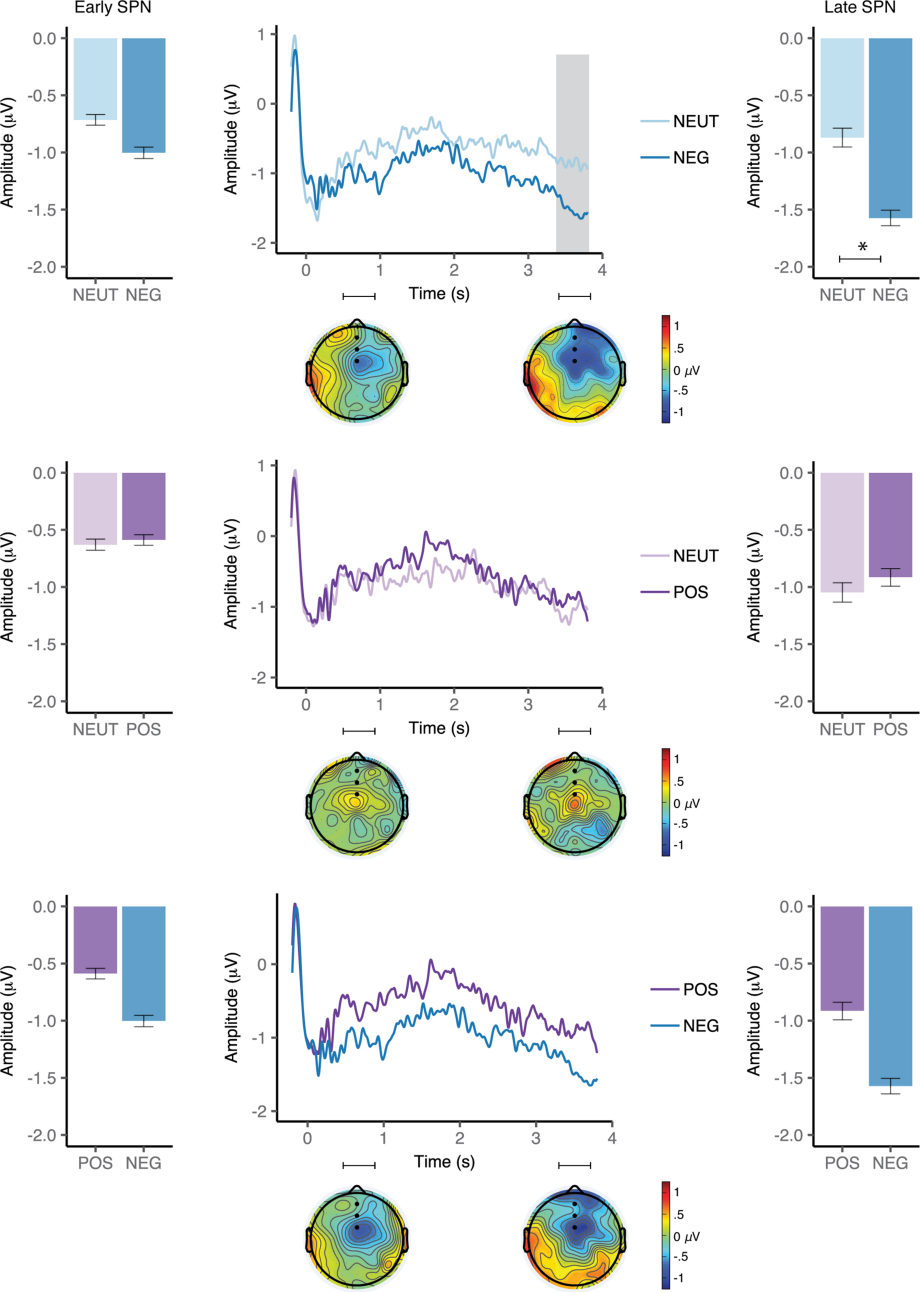
Event-related potentials elicited by neutral and negative (top), neutral and positive (middle) and positive and negative (bottom) English word cues in the monolingual group. Waveforms illustrate brain potential variations computed via linear derivation from three midline frontal electrodes (AFZ, FZ and FCZ). The window of analysis started just after verbal cue presentation, coinciding with the display of a fixation cross. The shaded area highlights the window of significant differences. Black dots on the topographical maps depict the electrode sites of interest. Bar graphs represent mean amplitude averaged over the electrodes of interest in each condition in the early (left panel) and late (right panel) SPN window. Error bars depict 95% confidence interval.

***SPN***. We found no significant differences between negative (*M* = −2.88 μV, 95% CI [−4.32, −1.45]) and neutral (*M* = −1.97 μV, 95% CI [−3.45, −0.49]) cue conditions in the early SPN time window, *t*(17) = −1.4, *P* = 0.18, Cohen’s *d* = 0.32. In the late window, SPN amplitude was more pronounced in the anticipation of a picture cued by a negative (*M* = −1.53 μV, 95% CI [−2.03, –1.03]) than a neutral (*M* = −0.81 μV, 95% CI [−1.62, −0.01]) word (*t*(17) = −2.57, *P* = 0.02, Cohen’s *d* = 0.61; Figure 6).

**Fig. 7:**
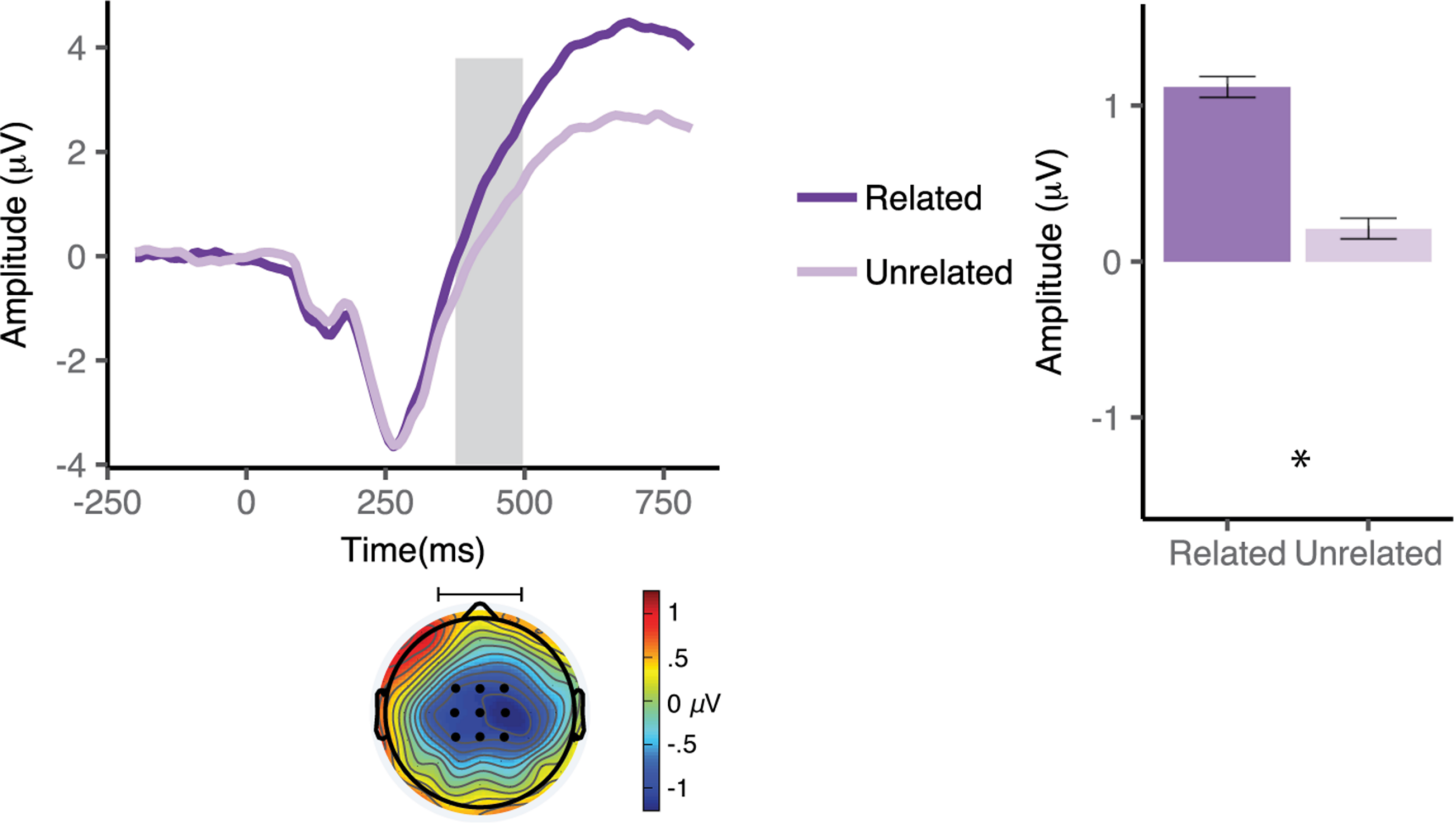
ERPs elicited by picture targets (S2) as a function of relatedness with the preceding verbal cue (S1) in the monolingual group. Waveforms illustrate brain potential variations computed via linear derivation from nine centro–parietal electrodes (FC1, FCz, FC2, C1, Cz, C2, CP1, CPZ and CP2). Time 0 coincides with picture presentation onset. Shaded gray areas highlight the window(s) of significant differences. Black dots on the topographical maps depict the electrode sites of interest. The bar graph represents mean N400 amplitude averaged over the electrodes of interest in the window of analysis. Error bars depict 95% confidence interval.

***N400***. The RM ANOVA revealed a significant main effect of relatedness, *F*(1,16) = 21.1, *P* < 0.001, ${\eta}_{\mathrm{p}}^2$ = 0.57, the N400 being more negative in response to pictures unrelated to the cue (*M* = 0.18 μV, 95% CI [−0.73, 1.1]) than those related to the cue (*M* = 1.1 μV, 95% CI [0.3, 2.0]; Figure 7). Neither the main effect of valence nor the relatedness × valence interaction turned out to be significant (*P* > 0.05). In the similar vein to the bilingual group, N400 topography was asymmetric, with slightly greater amplitudes over the right hemisphere.

## Discussion

We investigated whether the anticipation of emotional information is contingent upon the language of operation in bilinguals. We hypothesized that anticipating a negative picture cued by a negative word in L1 would lead to more pronounced amplitudes of the early and late SPN than when cued by a neutral word. Moreover, such difference would be significantly reduced or even fail to reach significance in L2. Electrophysiological data supported our hypothesis, showing increased amplitudes in the early and late SPN window in the anticipation of pictures cued by negative words in the bilinguals’ L1 (Polish) but not in their L2 (English). As expected, the same English words with a negative valence elicited enhanced SPN amplitudes in the monolingual English control group, the effect being restricted to the late SPN window. However, the predicted differences in the bilingual group occurred in the absence of a significant critical interaction between language and valence.

We contend that the absence of an interaction between language and valence in bilinguals relates to three characteristics of the present study that should be considered in the planning of future experiments. First, online activation of translation equivalents is likely to have played a role in reducing the size of such a potential interaction, given that access to translation equivalents has been shown to be unconscious and automatic in sequential bilinguals (e.g. Thierry and Wu, 2007; Wu and Thierry, 2010). Even though such effects have also been shown to be reduced for negatively valenced words (Wu and Thierry, 2012), it must be kept in mind that the bilinguals involved in the current study were highly proficient in English and immersed in an English speaking environment for an average of 6 years (range 3 months—16 years). This means that language non-selective lexical access might have been more effective in the Polish–English bilinguals tested here than in a group of Chinese–English bilinguals having spent only a few months in the UK, for instance. And, indeed, the valence effect observed in bilinguals in the English cueing blocks was close to marginal (*P* = 0.08). In other words, while the interaction may have been measurable in less balanced bilinguals or using a different paradigm (Jończyk *et al.,* 2016), it may well be that non-selective language access in the current sample was too strong to allow the interaction to appear. In addition, the affective pictures selected for this study were particularly mild (see detailed discussion of this point below) compared with the kind of highly arousing stimuli used in SPN experiments previously (e.g. from the IAPS database, Lang *et al.,* 1997). Finally, the experiment involved a high-level of stimulus repetition (picture targets were seen four times each by bilingual participants), which will have likely increased habituation effect (a factor known to reduce SPN sensitivity, Catena *et al.,* 2012).

Taken together, our findings corroborate previous evidence showing that linguistic cues, particularly those of negative valence, can elicit affective anticipation effects (Swannell *et al.,* 2016). Furthermore, this effect could be reduced in the L2 of bilinguals, suggesting that language may act as a modulating factor of affective anticipation (see Poli *et al.,* 2007; Brunia *et al.,* 2011a; Swannell *et al.,* 2016; Kotani *et al.,* 2017). Future studies using less balanced bilinguals and more emotionally potent stimuli will hopefully confirm such modulation or discard it more clearly.

Previous studies on physiological anticipation often measured SPN at the FZ electrode site only (Moser *et al.,* 2009; Thiruchselvam *et al.,* 2011; Fuentemilla *et al.,* 2013; Morís *et al.,* 2013; Pornpattananangkul *et al.,* 2017). Others reported more extensive scalp distribution covering the midline electrode sites (FPZ, FZ, FCz, Cz, CPZ, PZ, POZ; Poli *et al.,* 2007). Here, we report a somewhat intermediate result, with SPN amplitude being maximal over AFZ, FZ and FCz. At least two methodological choices in the current experiment might have worked against a more widespread effect.

First, we elected not to rely on frequently used pictorial stimuli from affective picture databases (e.g. Lang *et al.,* 1997; Dan-Glauser and Scherer, 2011; Marchewka *et al.,* 2014), because we considered such pictures too arousing and potentially disturbing, or in some cases even traumatizing. Beyond the potential ethical issues arising from the use of such stimuli, we made this choice because we wanted our results to be more ecologically valid and more representative of everyday functioning of the human brain. Indeed, one rarely witnesses lethal accidents or mutilations, and thus, results from studies that have used emotionally extreme stimuli might have artificially inflated the emotional response or even elicited responses that are not the representative of everyday experience. Affective valence and arousal were, therefore, low in this experiment, as compared with the literature and thus the anticipation effects measured could be expected to be somewhat weaker. If the goal of future experiments is to establish reduced sensitivity in the second language to potentially traumatic stimuli, then experimenters might be advised to use far more potent affective materials.

Second, we used a fully rotated, counterbalanced experimental design likely to have further weakened the strength of anticipatory processes. While this avoided the need for verbal cue repetition in each of the languages, picture repetition might have reduced SPN amplitudes through habituation. A few of the bilingual participants indicated that they had been able to guess the upcoming picture in some cases in the third and/or fourth experimental blocks. Given that the experimental sessions were approximately twice as long in the bilingual group, and participants ended up being exposed to more repetition, which might have in turn reduced the anticipation effect to a greater extent. An alternative explanation is that our groups differed in affective suggestiveness. Future studies could use yoked control groups to test this possibility and more detailed psychometric measures may be needed to tap into between-group differences in general emotional sensitivity. Nevertheless, taking into account that the anticipation of uncertain outcomes tends to yield more intense experience (Catena *et al.,* 2012) and considering that SPN amplitude tends to decrease as a function of learning (Morís *et al.,* 2013), a potential valence-dependent effect of language on SPN amplitude could have been weakened in this study.

In line with previous studies of semantic processing in bilinguals, we found that SPN amplitudes were overall greater after an English than a Polish cue, which could be due to a partial overlap with the N400 component elicited by the cue, and generally consistent with the idea that processing information in the second language tends to require greater cognitive effort (Frenck-Mestre, 2002; Moreno and Kutas, 2005; Thierry and Wu, 2007; Martin *et al.,* 2013). However, both early and late SPN modulations were observed well beyond the window of semantic processing, lending support to the fact that they did reflect anticipation of the target picture.

Moreover, we observed a language main effect in bilinguals, which could be interpreted as a sign of greater overall anticipation in L2 than in L1 or as a sign that when participants were exposed to L2 word cues, their L1 was supressed to a greater extent than their L2 when they were exposed to L1 word cues (an effect akin to that shown by Wu and Thierry, 2017, in the context of picture naming). If this differential suppression account is correct, then both negative and neutral words may have led to the inhibition of L1 when cues were in L2, with L2 inhibition being weaker for neutral than negative words when cues were in L1. Even though this leads to a slightly different interpretation of the results, this account remains consistent with greater affective sensitivity in L1 than L2.

Our findings suggest that highly proficient and immersed bilinguals operating in their second language may experience a reduction in sensitivity when anticipating affective content. This potential reduction, however, failed to yield a critical interaction in the current study unlike in previous studies showing that proficient bilinguals are less reactive to negative information presented in the second language and appear ‘protected’ against its negative impact. For instance, in the study by Wu and Thierry (2012), Chinese–English bilinguals did not show a predicted priming effect relating to unconscious non-selective lexical access from English to Chinese (Thierry and Wu, 2007; Wu and Thierry, 2010) when the English prime word had a negative valence. In a more recent study using emotionally realistic sentence contexts, access to negative information in the second language of immersed, proficient Polish–English bilinguals appeared to be reduced (Jończyk *et al.,* 2016). Furthermore, converging observations based on pupil dilation (Iacozza *et al.,* 2017) and electrodermal measurements (Jankowiak and Korpal, 2017; see also García-Palacios *et al.,* 2018) lend support to the idea that individuals are more detached from emotionally charged information in the L2 (for a discussion of affective disembodiment in bilingualism, see Pavlenko, 2012; Jończyk, 2016c; Sheikh and Titone, 2016), at the same time providing neurophysiological support for findings reported in introspective and clinical bilingual contexts (see Pavlenko, 2012; Caldwell-Harris, 2015; Jończyk, 2016a).

Despite the lack of a significant language by valence interaction in our bilingual group, our findings within each language context are not inconsistent with the so-called ‘foreign language effect’ (Keysar *et al.,* 2012; Costa *et al.,* 2014a). When bilinguals operate in their second language, they have been shown to exhibit a more utilitarian behavior (Costa *et al.,* 2014b; Geipel *et al.,* 2015; Corey *et al.,* 2017; Costa *et al.,* 2017). Furthermore, late bilinguals are willing to take more risks when receiving positive feedback in their native language, that is a reduction of the hot-hand effect when they operate in their second language (Gao *et al.,* 2015). The foreign language effect is thought to relate to the subjective impression of relative affective detachment when one operates in the second language, in line with hypotheses made on the basis of introspective approaches to bilingualism and emotion (for recent reviews, see Dewaele, 2010; Caldwell-Harris, 2015; Jończyk, 2016a) and bilinguals’ autobiographical memory (Schrauf and Rubin, 1998, 2000; Larsen *et al.,* 2002; Marian and Kaushanskaya, 2008; Pavlenko, 2014).

## Conclusion

In this study, we looked into the effects of language of operation on the anticipation of a forthcoming emotional picture in a classical S1–S2 priming paradigm. ERP data provided evidence regarding the feasibility of a neurophysiological investigation of affective anticipation in bilinguals, given that a reliable SPN modulation was found in both bilinguals and monolinguals when they operated in their native language. Furthermore, our results suggest that emotional experience in the second language may be reduced in power, although this would require validation based on a within-subject interaction between affective valence and language of operation. Given the fact that participants in our study were very fluent in English and immersed in the L2 culture for a significant period of time, we believe that this difference may be more pronounced in less experienced bilinguals. Also, increasing the potency of emotional stimuli may lead to a significant within subject effect, but we are conscious that this may come at the cost of ecological validity.

### Code availability

All MATLAB code used for the pre-processing of the data is available at Open Science Framework (OSF), https://osf.io/tdzsk/.

### Data availability

The data collected and analyzed for the purpose of the current study is available from the corresponding author upon reasonable request.

## Notes

^†^Rafał Jończyk and Guillaume Thierry designed research. Rafał Jończyk, Inga Korolczuk and Evangelia Balatsou collected the data. Rafał Jończyk and Guillaume Thierry analyzed the data. Rafał Jończyk, Inga Korolczuk, Evangelia Balatsou and Guillaume Thierry wrote the paper.

## References

[ref1] Bar M. (2007). The proactive brain: using analogies and associations to generate predictions. Trends in Cognitive Sciences, 11(7), 280–9. 17548232 10.1016/j.tics.2007.05.005

[ref2] Bar M. (2009). The proactive brain: memory for predictions. Philosophical Transactions of the Royal Society of London. Series B, Biological Sciences, 364(1521), 1235–43. doi: 10.1098/rstb.2008.0310. 19528004 PMC2666710

[ref3] Barrett L.F., Bar M. (2009). See it with feeling: affective predictions during object perception. Philosophical Transactions of the Royal Society of London. Series B, Biological Sciences, 364(1521), 1325–34. doi: 10.1098/rstb.2008.0312. 19528014 PMC2666711

[ref4] Barrett L.F., Simmons W.K. (2015). Interoceptive predictions in the brain Nature Reviews. Neuroscience, 16(7), 419–29. 26016744 10.1038/nrn3950 PMC4731102

[ref5] Baumeister J.C., Foroni F., Conrad M., Rumiati R.I., Winkielman P. (2017). Embodiment and emotional memory in first vs. second language. Frontiers in Psychology, 8(351), 394. doi: 10.3389/fpsyg.2017.00394. 28386240 PMC5362726

[ref6] Böcker K.B., Baas J.M., Kenemans J.L., Verbaten M.N. (2001). Stimulus-preceding negativity induced by fear: a manifestation of affective anticipation. International Journal of Psychophysiology: Official Journal of the International Organization of Psychophysiology, 43(1), 77–90. 11742686 10.1016/s0167-8760(01)00180-5

[ref7] van Boxtel G.J.M., Böcker K.B.E. (2004). Cortical measures of anticipation. Journal of Psychophysiology, 18(2/3), 61–76. doi: 10.1027/0269-8803.18.23.61.

[ref8] Brunia C.H.M., van Boxtel G.J.M. (2004). Anticipatory attention to verbal and non-verbal stimuli is reflected in a modality-specific SPN. Experimental Brain Research, 156(2), 231–9. doi: 10.1007/s00221-003-1780-2. 15344853

[ref9] Brunia C.H.M., Hackley S.A., van Boxtel G.J.M., Kotani Y., Ohgami Y. (2011a). Waiting to perceive: reward or punishment? Clinical Neurophysiology: Official Journal of the International Federation of Clinical Neurophysiology, 122(5), 858–68. doi: 10.1016/j.clinph.2010.12.039 21215692

[ref10] Brunia C.H.M., van Boxtel G.J.M., Böcker K.B.E. (2012). Negative slow waves as indices of anticipation: The bereitschaftspotential, the contingent negative variation, and the stimulus-preceding negativity In: Luck SJ, Kappenman ES, editors. The Oxford handbook of event-related potential components. Oxford: Oxford University Press 189–207.

[ref11] Caldwell-Harris C.L. (2015). Emotionality differences between a native and foreign language: implications for everyday life. Current Directions in Psychological Science, 24(3), 214–9. doi: 10.1177/0963721414566268.

[ref15] Catena A., Perales J.C., Megías A., Cándido A., Jara E., Maldonado A. (2012). The brain network of expectancy and uncertainty processing. PLoS One, 7(7), e40252. doi: 10.1371/journal.pone.0040252. 22768344 PMC3388057

[ref16] Conrad M., Recio G., Jacobs A.M. (2011). The time course of emotion effects in first and second language processing: a cross cultural ERP study with German-Spanish bilinguals. Frontiers in Psychology, 2, 351. doi: 10.3389/fpsyg.2011.00351. 22164150 PMC3230907

[ref17] Corey J.D., Hayakawa S., Foucart A., Aparici M., Botella J., Costa A., Keysar B. (2017). Our moral choices are foreign to us. Journal of Experimental Psychology. Learning, Memory, and Cognition, 43(7), 1109–28. doi: 10.1037/xlm0000356. 28068125

[ref18] Costa A., Foucart A., Arnon I., Aparici M., Apesteguia J. (2014a). ‘Piensa’ twice: on the foreign language effect in decision making. Cognition, 130(2), 236–54. doi: 10.1016/j.cognition.2013.11.010. 24334107

[ref19] Costa A., Foucart A., Hayakawa S., Aparici M., Apesteguia J., Heafner J., Keysar B. (2014b). Your morals depend on language. PLOS One, 9(4), e94842. doi: 10.1371/journal.pone.0094842 24760073 PMC3997430

[ref20] Costa A., Vives M., Corey J.D. (2017). On language processing shaping decision making. Current Directions in Psychological Science, 26(2), 146–51. doi: 10.1177/0963721416680263.

[ref23] Dan-Glauser E.S., Scherer K.R. (2011). The Geneva affective picture database (GAPED): a new 730-picture database focusing on valence and normative significance. Behavior Research Methods, 43(2), 468–77. doi: 10.3758/s13428-011-0064-1. 21431997

[ref24] Degner J., Doycheva C., Wentura D. (2012). It matters how much you talk: on the automaticity of affective connotations of first and second language words. Bilingualism: Language and Cognition, 15(1), 181–9.

[ref25] Delorme A., Makeig S. (2004). EEGLAB: an open source toolbox for analysis of single-trial EEG dynamics including independent component analysis. Journal of Neuroscience Methods, 134(1), 9–21. 15102499 10.1016/j.jneumeth.2003.10.009

[ref28] Dewaele J.-M. (2010). Emotions in multiple languages In: Houndmills, Basingstoke, Hampshire, New York, NY: Palgrave Macmillan.

[ref29] Eilola T.M., Havelka J., Sharma D. (2007). Emotional activation in the first and second language. Cognition & Emotion, 21(5), 1064–76. doi: 10.1080/02699930601054109.

[ref30] Engel A.K., Fries P., Singer W. (2001). Dynamic predictions: oscillations and synchrony in top-down processing. Nature Reviews Neuroscience, 2(10), 704–16. doi: 10.1038/35094565. 11584308

[ref31] Ferré P., García T., Fraga I., Sánchez-Casas R., Molero M. (2010). Memory for emotional words in bilinguals: do words have the same emotional intensity in the first and in the second language? Cognition & Emotion, 24(5), 760–85. doi: 10.1080/02699930902985779.

[ref32] Ferré P., Sánchez-Casas R., Fraga I. (2013). Memory for emotional words in the first and the second language: effects of the encoding task. Bilingualism: Language and Cognition, 16(3), 495–507. doi: 10.1017/S1366728912000314.

[ref34] Frenck-Mestre C. (2002). An on-line look at sentence processing in the second language In: Heredia R.R., Altarriba J., editors. Bilingual Sentence Processing, Vol. 134, Amsterdam, Netherlands: North-Holland/Elsevier Science Publishers, pp. 217–36.

[ref35] Fuentemilla L., Cucurell D., Marco-Pallarés J., Guitart-Masip M., Morís J., Rodríguez-Fornells A. (2013). Electrophysiological correlates of anticipating improbable but desired events. Neuro Image, 78, 135–44. doi: 10.1016/j.neuroimage.2013.03.062. 23583745

[ref36] Gao S., Zika O., Rogers R.D., Thierry G. (2015). Second language feedback abolishes the ‘hot hand’ effect during even-probability gambling. The Journal of Neuroscience, 35(15), 5983–9. doi: 10.1523/JNEUROSCI.3622-14.2015. 25878271 PMC4397599

[ref37] García-Palacios A., Costa A., Castilla D., Río E., Casaponsa A., Duñabeitia J.A. (2018). The effect of foreign language in fear acquisition. Scientific Reports, 8(1), 1157. doi: 10.1038/s41598-018-19352-8. 29348683 PMC5773680

[ref38] Geipel J., Hadjichristidis C., Surian L. (2015). How foreign language shapes moral judgment. Journal of Experimental Social Psychology, 59, 8–17.

[ref39] Grabovac B., Pléh C. (2014). Emotional activation measured using the emotional Stroop task in early Hungarian-Serbian bilinguals from Serbia. Acta Linguistica Hungarica, 61(4), 423–41. doi: 10.1556/ALing.61.2014.4.3.

[ref41] van Heuven W.J.B., Mandera P., Keuleers E., Brysbaert M. (2014). SUBTLEX-UK: a new and improved word frequency database for British English. Quarterly Journal of Experimental Psychology, 67(6), 1176–90. doi: 10.1080/17470218.2013.850521. 24417251

[ref42] Hoemann K., Gendron M., Barrett L.F. (2017). Mixed emotions in the predictive brain. Current Opinion in Behavioral Sciences, 15, 51–7. doi: 10.1016/j.cobeha.2017.05.013. 29109966 PMC5669377

[ref43] Hsu C.-T., Jacobs A.M., Conrad M. (2015). Can Harry potter still put a spell on us in a second language? An fMRI study on reading emotion-laden literature in late bilinguals. Cortex: a Journal Devoted to the Study of the Nervous System and Behavior, 63, 282–95. doi: 10.1016/j.cortex.2014.09.002. 25305809

[ref44] Iacozza S., Costa A., Duñabeitia J.A. (2017). What do your eyes reveal about your foreign language? Reading emotional sentences in a native and foreign language. PLoS One, 12(10), e0186027. doi: 10.1371/journal.pone.0186027. 28973016 PMC5626519

[ref45] Jankowiak K., Korpal P. (2017). On modality effects in bilingual emotional language processing: evidence from galvanic skin response. Journal of Psycholinguistic Research. 47(3), 663–677. doi: 10.1007/s10936-017-9552-5. PMC5937920 29285592

[ref46] Jończyk R. (2016a). Affect-language interactions in nonnative speakers In: Jończyk R., editors. Affect-Language Interactions in Native and Non-Native English Speakers (pp. 75–101). Cham: Springer International Publishing Retrieved from http://link.springer.com/10.1007/978-3-319-47635-3_4

[ref47] Jończyk R. (2016b). Affective word processing in native and nonnative english speakers: a neuropragmatic perspective In: Jończyk R., editor. Affect-Language Interactions in Native and Non-Native English Speakers (pp. 103–31). Cham: Springer International Publishing Retrieved from http://link.springer.com/10.1007/978-3-319-47635-3_5

[ref48] Jończyk R. (2016c). Affective (dis) embodiment in nonnative language In Jończyk R., editor. Affect-Language Interactions in Native and Non-Native English Speakers (pp. 149–59). Cham: Springer International Publishing Retrieved from http://link.springer.com/10.1007/978-3-319-47635-3_7

[ref49] Jończyk R., Boutonnet B., Musiał K., Hoemann K., Thierry G. (2016). The bilingual brain turns a blind eye to negative statements in the second language. Cognitive, Affective, & Behavioral Neuroscience. 10.3758/s13415-016-0411-x PMC4868866 26926623

[ref51] Keysar B., Hayakawa S.L., An S.G. (2012). The foreign-language effect thinking in a foreign tongue reduces decision biases. Psychological Science, 23(6), 661–8. 22517192 10.1177/0956797611432178

[ref52] Kotani Y., Hiraku S., Suda K., Aihara Y. (2001). Effect of positive and negative emotion on stimulus-preceding negativity prior to feedback stimuli. Psychophysiology, 38(6), 873–8. 12240663 10.1111/1469-8986.3860873

[ref53] Kotani Y., Ohgami Y., Yoshida N., Kiryu S., Inoue Y. (2017). Anticipation process of the human brain measured by stimulus-preceding negativity (SPN). The Journal of Physical Fitness and Sports Medicine, 6(1), 7–14. doi: 10.7600/jpfsm.6.7.

[ref54] Kutas M., Federmeier K.D. (2011). Thirty years and counting: finding meaning in the N400 component of the event-related brain potential (ERP). Annual Review of Psychology, 62, 621–47. doi: 10.1146/annurev.psych.093008.131123. PMC4052444 20809790

[ref55] Lang P.J., Bradley M.M., Cuthbert B.N. (1997). International Affective Picture System (IAPS): Technical Manual and Affective Ratings, NIMH Center for the Study of Emotion and Attention, pp. 39–58.

[ref56] de Lange F.P., Fritsche M. (2017). Perceptual decision-making: picking the low-hanging fruit? Trends in Cognitive Sciences, 21(5), 306–7. doi: 10.1016/j.tics.2017.03.006. 28343760

[ref57] Larsen S.F., Schrauf R.W., Fromholt P., Rubin D.C. (2002). Inner speech and bilingual autobiographical memory: a polish-Danish cross-cultural study. Memory, 10(1), 45–54. doi: 10.1080/09658210143000218. 11747575

[ref58] Lee T.-W., Girolami M., Sejnowski T.J. (1999). Independent component analysis using an extended infomax algorithm for mixed subgaussian and supergaussian sources. Neural Computation, 11(2), 417–41. doi: 10.1162/089976699300016719. 9950738

[ref59] Li P., Zhang F., Tsai E., Puls B. (2014). Language history questionnaire (LHQ 2.0): a new dynamic web-based research tool. Bilingualism: Language and Cognition, 17(3), 673–80. doi: 10.1017/S1366728913000606.

[ref60] Lopez-Calderon J., Luck S.J. (2014). ERPLAB: an open-source toolbox for the analysis of event-related potentials. Frontiers in Human Neuroscience, 8. doi: 10.3389/fnhum.2014.00213. 24782741 PMC3995046

[ref61] Mandera P., Keuleers E., Wodniecka Z., Brysbaert M. (2015). Subtlex-pl: subtitle-based word frequency estimates for polish. Behavior Research Methods, 47(2), 471–83. doi: 10.3758/s13428-014-0489-4. 24942246

[ref62] Marchewka A., Żurawski Ł., Jednoróg K., Grabowska A. (2014). The Nencki affective picture system (NAPS): introduction to a novel, standardized, wide-range, high-quality, realistic picture database. Behavior Research Methods, 46(2), 596–610. doi: 10.3758/s13428-013-0379-1. 23996831 PMC4030128

[ref63] Marian V., Kaushanskaya M. (2008). Words, feelings, and bilingualism. The Mental Lexicon, 3(1), 72–90. doi: 10.1075/ml.3.1.06mar. 19966924 PMC2788822

[ref64] Martin C.D., Thierry G., Kuipers J.-R., Boutonnet B., Foucart A., Costa A. (2013). Bilinguals reading in their second language do not predict upcoming words as native readers do. Journal of Memory and Language, 69(4), 574–88. doi: 10.1016/j.jml.2013.08.001.

[ref65] Michalowski J.M., Pané-Farré C.A., Löw A., Hamm A.O. (2015). Brain dynamics of visual attention during anticipation and encoding of threat- and safe-cues in spider-phobic individuals. Social Cognitive and Affective Neuroscience, 10(9), 1177–86. doi: 10.1093/scan/nsv002. 25608985 PMC4560937

[ref66] Moreno E.M., Kutas M. (2005). Processing semantic anomalies in two languages: an electrophysiological exploration in both languages of Spanish-English bilinguals. Brain Research. Cognitive Brain Research, 22(2), 205–20. doi: 10.1016/j.cogbrainres.2004.08.010. 15653294

[ref67] Morís J., Luque D., Rodríguez-Fornells A. (2013). Learning-induced modulations of the stimulus-preceding negativity: learning modulation of SPN. Psychophysiology, 50(9), 931–9. doi: 10.1111/psyp.12073. 23808750

[ref68] Moser J.S., Krompinger J.W., Dietz J., Simons R.F. (2009). Electrophysiological correlates of decreasing and increasing emotional responses to unpleasant pictures. Psychophysiology, 46(1), 17–27. doi: 10.1111/j.1469-8986.2008.00721.x. 18992073

[ref69] Ohgami Y., Kotani Y., Arai J.-I., Kiryu S., Inoue Y. (2014). Facial, verbal, and symbolic stimuli differently affect the right hemisphere preponderance of stimulus-preceding negativity. Psychophysiology, 51(9), 843–52. doi: 10.1111/psyp.12234. 24849660

[ref70] Opitz B., Degner J. (2012). Emotionality in a second language: it’s a matter of time. Neuropsychologia, 50(8), 1961–7. doi: 10.1016/j.neuropsychologia.2012.04.021. 22569217

[ref71] Pavlenko A. (2012). Affective processing in bilingual speakers: disembodied cognition? International Journal of Psychology: Journal International De Psychologie, 47(6), 405–28. doi: 10.1080/00207594.2012.743665. 23163422

[ref72] Pavlenko A. (2014). The Bilingual Mind: And What It Tells Us About Language And Thought, Cambridge, New York: Cambridge University Press.

[ref73] Pei G., Meng L. (2016). What do we expect from a beauty? Facial attractiveness of the opposite sex gives rise to discrepancies in males’ anticipation and demand. International Journal of Psychology: Journal International De Psychologie, 8, 537. doi: 10.1002/ijop.12393. 27658937

[ref74] Perri R.L., Berchicci M., Lucci G., Cimmino R.L., Bello A., Di Russo F. (2014). Getting ready for an emotion: specific premotor brain activities for self-administered emotional pictures. Frontiers in Behavioral Neuroscience, 8, 197. doi: 10.3389/fnbeh.2014.00197. 24904344 PMC4035832

[ref75a] Pion-Tonachini L., Kreutz-Delgado K., Makeig S. (2019). ICLabel: An automated electroencephalographic independent component classifier, dataset, and website. NeuroImage, 198, 181–197. doi: 10.1016/j.neuroimage.2019.05.026. 31103785 PMC6592775

[ref75] Poli S., Sarlo M., Bortoletto M., Buodo G., Palomba D. (2007). Stimulus-preceding negativity and heart rate changes in anticipation of affective pictures. International Journal of Psychophysiology: Official Journal of the International Organization of Psychophysiology, 65(1), 32–9. doi: 10.1016/j.ijpsycho.2007.02.008. 17395326

[ref78] Pornpattananangkul N., Nadig A., Heidinger S., Walden K., Nusslock R. (2017). Elevated outcome-anticipation and outcome-evaluation ERPs associated with a greater preference for larger-but-delayed rewards. Cognitive, Affective, & Behavioral Neuroscience, 17(3), 625–41. doi: 10.3758/s13415-017-0501-4. PMC5409980 28224457

[ref79] Schrauf R.W., Rubin D.C. (1998). Bilingual autobiographical memory in older adult immigrants: a test of cognitive explanations of the reminiscence bump and the linguistic encoding of memories. Journal of Memory and Language, 39(3), 437–57. doi: 10.1006/jmla.1998.2585.

[ref80] Schrauf R.W., Rubin D.C. (2000). Internal languages of retrieval: the bilingual encoding of memories for the personal past. Memory & Cognition, 28(4), 616–23. doi: 10.3758/BF03201251. 10946544

[ref81] Seidel E.-M., Pfabigan D.M., Hahn A., Sladky R., Grahl A., Paul K., Lamm C. (2015). Uncertainty during pain anticipation: the adaptive value of preparatory processes. Human Brain Mapping, 36(2), 744–55. doi: 10.1002/hbm.22661. 25324216 PMC6869185

[ref82] Shafir R., Schwartz N., Blechert J., Sheppes G. (2015). Emotional intensity influences pre-implementation and implementation of distraction and reappraisal. Social Cognitive and Affective Neuroscience, 10(10), 1329–37. doi: 10.1093/scan/nsv022. 25700568 PMC4590533

[ref83] Sheikh N.A., Titone D. (2016). The embodiment of emotional words in a second language: an eye-movement study. Cognition & Emotion, 30(3), 488–500. doi: 10.1080/02699931.2015.1018144. 25786993

[ref85] Sterling P. (2012). Allostasis: a model of predictive regulation. Physiology & Behavior, 106(1), 5–15. doi: 10.1016/j.physbeh.2011.06.004. 21684297

[ref86] Summerfield C., de Lange F.P. (2014). Expectation in perceptual decision making: neural and computational mechanisms. Nature Reviews Neuroscience, 15(11), 745–56. doi: 10.1038/nrn3838. 25315388

[ref87] Sutton T.M., Altarriba J., Gianico J.L., Basnight-Brown D.M. (2007). The automatic access of emotion: emotional Stroop effects in Spanish–English bilingual speakers. Cognition & Emotion, 21(5), 1077–90. doi: 10.1080/02699930601054133.

[ref88] Swannell E.R., Brown C.A., Jones A.K.P., Brown R.J. (2016). Some words hurt more than others: semantic activation of pain concepts in memory and subsequent experiences of pain. The Journal of Pain: Official Journal of the American Pain Society, 17(3), 336–49. doi: 10.1016/j.jpain.2015.11.004. 26681115

[ref89] Takeuchi S., Mochizuki Y., Masaki H., Takasawa N., Yamazaki K. (2005). Stimulus preceding negativity represents arousal induced by affective picture. International Congress Series, 1278, 385–8. doi: 10.1016/j.ics.2004.11.135.

[ref90] Thierry G., Wu Y.J. (2007). Brain potentials reveal unconscious translation during foreign-language comprehension. Proceedings of the National Academy of Sciences, 104(30), 12530–5. doi: 10.1073/pnas.0609927104. PMC1941503 17630288

[ref91] Thiruchselvam R., Blechert J., Sheppes G., Rydstrom A., Gross J.J. (2011). The temporal dynamics of emotion regulation: an EEG study of distraction and reappraisal. Biological Psychology, 87(1), 84–92. doi: 10.1016/j.biopsycho.2011.02.009. 21354262

[ref92] Van Berkum J.J.A. (2010). The brain is a prediction machine that cares about good and bad—any implications for neuropragmatics. Italian Journal of Linguistics, 22(1), 181–208.

[ref93] Van Berkum J.J.A. (2013). Anticipating communication. Theoretical Linguistics, 39(1/2), 75–86. doi: 10.1515/tl-2013-0004.

[ref95] Walter W.G., Cooper R., Aldridge V.J., McCallum W.C., Winter A.L. (1964). Contingent negative variation: an electric sign of sensori-motor association and expectancy in the human brain. Nature, 203(4943), 380–4. doi: 10.1038/203380a0. 14197376

[ref96] Warriner A.B., Kuperman V., Brysbaert M. (2013). Norms of valence, arousal, and dominance for 13,915 English lemmas. Behavior Research Methods, 45(4), 1191–207. doi: 10.3758/s13428-012-0314-x. 23404613

[ref97] Winskel H. (2013). The emotional Stroop task and emotionality rating of negative and neutral words in late Thai-English bilinguals. International Journal Of Psychology: Journal International De Psychologie, 48(6), 1090–8. doi: 10.1080/00207594.2013.793800. 23710830

[ref98a] Wu Y.J., Thierry G. (2010). Chinese-English bilinguals reading English hear Chinese. The Journal of Neuroscience: the Official Journal of the Society for Neuroscience, 30(22), 7646–7651. doi: 10.1523/JNEUROSCI.1602-10.2010. 20519539 PMC6632379

[ref98] Wu Y.J., Thierry G. (2012). How Reading in a second language protects your heart. The Journal of Neuroscience, 32(19), 6485–9. doi: 10.1523/JNEUROSCI.6119-11.2012. 22573670 PMC6621108

[ref99] Wu Y.J., Thierry G. (2017). Brain potentials predict language selection before speech onset in bilinguals. Brain and Language, 171, 23–30. doi: 10.1016/j.bandl.2017.04.002. 28445784

[ref100] Zheng Y., Liu X. (2015). Blunted neural responses to monetary risk in high sensation seekers. Neuropsychologia, 71, 173–80. doi: 10.1016/j.neuropsychologia.2015.04.002. 25843768

