# Erratum to: Keep calm and carry on: electrophysiological evaluation of emotional anticipation in the second language

**DOI:** 10.1093/scan/nsz102

**Published:** 2020-01-27

**Authors:** Rafał Jończyk, Inga Korolczuk, Evangelia Balatsou, Guillaume Thierry

**Affiliations:** Faculty of English, Adam Mickiewicz University, 60-780, Poznan, Poland; Department of Psychology, Pennsylvania State University, 16801, University Park, PA, USA; School of Psychology, Bangor University, LL57 2AS, Bangor, UK; School of Psychology, Bangor University, LL57 2AS, Bangor, UK; School of Psychology, Bangor University, LL57 2AS, Bangor, UK; Centre for Research on Bilingualism, Bangor University, LL57 2DG, Bangor, UK

The first version of this article displayed Figure 5 incorrectly. This has now been corrected. The publisher apologizes for the error.

